# A supramolecular approach towards strong and tough polymer nanocomposite fibers†

**DOI:** 10.1039/c8ra01066h

**Published:** 2018-03-14

**Authors:** Xiaojuan Zhao, Hongzhi Zheng, Dan Qu, Haijing Jiang, Wei Fan, Yuyuan Sun, Yan Xu

**Affiliations:** State Key Lab of Inorganic Synthesis and Preparative Chemistry, Jilin University 2699 Qianjin Street Changchun 130012 China yanxu@jlu.edu.cn +86 431 85168607

## Abstract

Polymer nanocomposite fibers are important one-dimensional nanomaterials that hold promising potential in a broad range of technological applications. It is, however, challenging to organize advanced polymer nanocomposite fibers with sufficient mechanical properties and flexibility. Here, we demonstrate that strong, tough and flexible polymer nanocomposite fibers can be approached by electrospinning of a supramolecular ensemble of dissimilar and complementary components including flexible multiwalled carbon nanotubes (CNT), and stiff cellulose nanocrystals (CNC) in an aqueous poly(vinyl alcohol) (PVA) system. CNT and CNC are bridged by a water-soluble aggregation-induced-emission (AIE) molecule that forms π–π stacking with CNT *via* its conjugated chains, and electrostatic attraction with CNC through its positive charges leading to a soluble CNT–AIE–CNC ensemble, which further assembles with PVA through hydrogen bonds. A high level of ordering of the nanoscale building blocks combined with hydrogen bonding leads to a more efficient stress transfer path between the reinforcing unit and the polymer. The nanocomposite fiber mat is capable of selective detection of nitroaromatic explosives.

## Abstract 2

Strong, tough and flexible polymer nanocomposite fibers can be approached by electrospinning of supramolecular ensemble of CNT–AIE–CNC/PVA in an aqueous system, which could be used for sensing.

## Graphical abstract

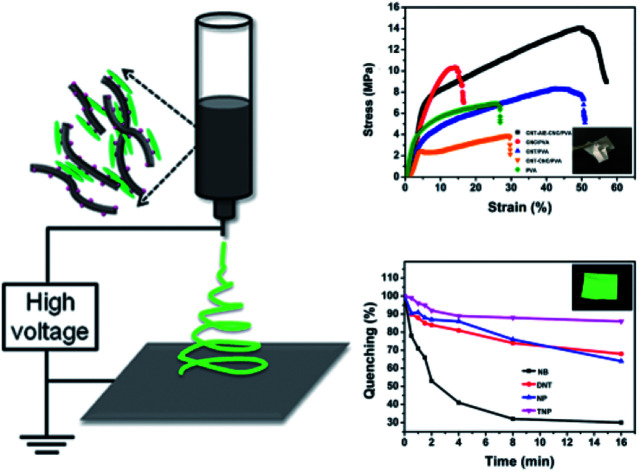

## Introduction

Electrospinning is a versatile, low-cost and scalable technique for producing high-aspect ratio fibers of a wide range of compositions with achievable diameters ranging from several nanometers to a few micrometers.^1,2^ The electrospun fiber formation process has the marked benefit that functional components are essentially confined in a nano–micro space along the fiber axis. By exploiting this technique, functional polymer nanocomposite fibers, such as carbon nanotube-reinforced poly(ethylene oxide), polyacrylonitrile and poly(methylmethacrylate),^3–5^ cellulose nanocrystal-reinforced poly(ethylene oxide)^6^ and poly(vinyl alcohol)^7^ have been approached by nanofilling.^8^ These nanocomposite fibers hold promising potentials as portable sensors,^9^ tissue engineering scaffolds,^10,11^ wearable energy devices,^12–14^ protective clothing^15^ and air filtration,^16^ however, their further applications are limited by their unsatisfactory mechanical properties (tensile strength, Young's modulus, elongation-at-break). Research shows that the mechanical properties of electrospun polymer fibers can be enhanced using nanoscale reinforcing fillers such as carbon nanotube (CNT, tensile strength ∼ 220 MPa) and sustainable cellulose nanocrystals CNC (CNC, tensile strength ∼ 7 GPa). For example, the tensile strength of CNT-reinforced polyacrylonitrile increased by 40% accompanied with 54% loss in the elongation-at-break compared to the electrospun fibers of pure polyacrylonitrile.^4^ The tensile strength of CNC-reinforced cellulose fiber mat showed 102% improvement while corresponding elongation-at-break showed 21% decrease compared to the pure electrospun polymer fiber mat.^17^ Recent efforts in developing biocompatible fiber mats for biological applications lead to a series of biocompatible polymer composite fibers from aqueous electrospinning systems, such as poly(ethylene oxide)- and poly(vinyl alcohol)-based (PEO- and PVA-based), however, random orientation of polymer chains within confined space render them poor mechanical properties.^18,19^ In an attempt to tackle the problem, Jiang and coworkers systematically investigated the reinforcing effect of CNC on the electrospun fibers of PEO.^20^ Han and coworkers further demonstrated that incorporation of CNC into PVA at low loading led to polymer nanocomposite fibers with improved tensile strength, unaltered Young's modulus and a slight drop in the elongation-at-break.^21^ Gholipourmalekabadi and coworkers show that significant improvement in the tensile strength, Young's modulus, and toughness was observed in chitosan-intercalated montmorillonite/poly(vinyl alcohol) fibers. The enhancement was attributed to the compounded weak interactions including hydrophobic–hydrophobic interactions, and intra- and inter-molecular hydrogen bonding between chitosan, montmorillonite and PVA in the blend. However, the elongations-at-break for all the fibers was very similar.^22^ Apparently, developing polymer nanocomposite fibers with balanced functional performance and satisfactory mechanical properties remains a challenge.

The deficiency in the mechanical properties of the electrospun composite fibers is largely associated with the ability to allow stress transfer across interfaces. To overcome these challenges, effective dispersion of reinforcing fillers at the nanoscale, alignment of polymer and reinforcing fillers along fiber axis, and bridging at filler–polymer interface and between reinforcing fillers are essential to allow effective load transfer.^23–25^ Here, we demonstrate a supermolecular approach towards control of the orientation, and the filler–filler and filler–polymer interfacial interactions at the nanoscale that can be retained at the macroscale in an aqueous system.

In pursuit of an effective combination of complementary components for advanced materials, we choose flexible and high aspect ratio pristine multiwalled carbon nanotube (CNT)^26^ and stiff nanorod-like CNC^27^ as reinforcing fillers, a positively charged water soluble salicylaldehyde azines derivatives (AIE) as a dispersing and bridging agent,^28^ and biocompatible and water soluble PVA as a representative polymer. Aggregation-induced emission (AIE)-based active materials was widely used as fluorescent probe for the detection of explosive.^29^ AIE agent used here is an electrostatic ensemble of salicyladazine fluorophore and positively charged pyridinium groups, denoted as AIE. We approach fabrication of polymer nanocomposite fibers from supramolecular ensemble of CNT–AIE–CNC/PVA in an aqueous system. Water insoluble CNT turns dispersible by forming CNT–AIE ensemble through π–π stacking, which further assemble with CNC leading to CNT–AIE–CNC ensemble through electrostatic attractions. The supramolecular ensemble of CNT–AIE–CNC interacts with PVA at the CNC end through multiple hydrogen bonds. The polymer nanocomposite fibers of CNT–AIE–CNC/PVA exhibit enhanced toughness, tensile strength, elongation-at-break and Young's modulus by 308%, 106%, 104% and 35%, respectively, compared with pure PVA electrospun fibers, owing to imposed alignment of CNT along the fiber axis, and the synergistic interactions between the fillers and at filler–polymer interface. The polymer nanocomposite fibers show promising potential as a portable fluorescent sensor for explosive detection. The current work suggests a facile and versatile strategy for rational design and organization of advanced polymer nanocomposite fibers from dissimilar and complementary components.

## Experimental

### Materials

Pristine multi-walled carbon nanotubes (AR, 10–20 nm in average diameter, 0.5–2 μm in average length, >98%) was purchased from Chengdu Organic Chemicals Co. Ltd. Cotton pulp board was purchased from Hebei Paper Group of China. Polyvinyl alcohol (AR, alcoholysis degree of 87–89%), 2,4-dihydroxybenzaldehyde (AR, 98%) and 1,6-dibromohexane (AR, 97%) were purchased from Aladdin Reagent (Shanghai) Co Ltd. Potassium carbonate (AR, 99%), hydrazine monohydrate (AR, 80%) and pyridine (AR, 99.5%) obtained from Guangfu Fine Chemicals. All reagents were used as received without further purification.

### Preparation

AIE and CNC were prepared according to reported procedures with the details, synthetic route and ^1^H NMR spectra given in the ESI (Fig. S1–4†).^29,30^

#### CNT–AIE ensemble

5 mg of CNT power was added to 5 mL of 0.3 wt% AIE aqueous solution, and the mixed solution was sonicated for 20 min and then stirred continuously for 24 h. The mixture was centrifuged at 13 000 rpm for 20 min and washed using ultrapure water (18.2 MΩ cm, Mill-Q Corp) to remove extra AIE. Next, the precipitate was dispersed in deionized water, denoted as CNT–AIE suspension, for further use.

#### CNT–AIE–CNC ensemble

A certain amount of CNC aqueous suspension (5.0 wt%) was added dropwise to the above CNT–AIE suspension solution under stirring. Then keep stirring for 24 h. A control was prepared by mechanical mixing of 1CNT : 3CNC (mass ratio) in an ultrasound bath, denoted as CNT–CNC.

#### CNT–AIE–CNC/PVA polymer nanocomposite fibers

A typical electrospinning solution was prepared as follows. 2.2 g of PVA aqueous solution (30 wt%) was added to a CNT–AIE–CNC suspension in stoichiometry. The PVA concentration was kept constant at 15 wt% for all preparations. The weight ratio of CNT–AIE–CNC in PVA solutions was varied to 0, 1, 3, 5 and 7 wt%. Electrospinning solutions containing CNT–AIE–CNC and PVA in stoichiometry was stirred for 24 h till a homogeneous mixture was obtained. The electrospinning solution was fed through a syringe with a stainless steel spinneret (inner diameter: 0.51 mm) under a voltage of 14–16 kV. Electrospinning was conducted at 20 °C and 30% relative humidity (RH), and the as-spun fibers were collected on grounded aluminum foil placed 20 cm below the tip of the spinneret. As-prepared fibers were dried in an oven at 30 °C for 24 h. Control fibers of CNC/PVA, CNT–AIE/PVA and CNT–CNC/PVA with the filler : polymer weight ratio of 3 : 97 were prepared under the same electrospinning conditions, designated as CNC/PVA, CNT–AIE/PVA and CNT–CNC/PVA, respectively.

### Characterization

Scanning electron microscopy (SEM, JEOL JSM 6700F), transmission electron microscopy (TEM, FEI Tecnai G2S-Twin with an EDS attachment), UV-visible spectroscopy (Shimadzu UV-1800), X-ray photoelectron spectroscopy (XPS, ESCALAB 250) and Elemental analysis (ElemenTar vario MICRO) were used. The electrophoretic mobility (*ζ*-potential) were measured using a Malvern Zetasizer Nano-ZS90. Photoemission spectra were recorded on a FLUORO, MAX-4 (JY American). Fourier transform infrared (FTIR) spectra were recorded using a BRUKER IFS-66V/S spectrometer in the frequency range of 400–4000 cm^−1^ with a resolution of 4 cm^−1^. Tensile tests were carried out on a universal material testing machine (Instron 5944, UK) equipped with a 100 N load cell. All measurements were conducted at room temperature, and specimens were conditioned at 25 °C and 50% RH for 24 h before testing. The specimen with approximate dimensions of ∼50 mm in length, ∼5 mm in width and ∼30 μm in thickness were tested for mechanical properties at a strain rate of 5 mm min^−1^ and a gauge length of 10 mm. 3–5 specimens were tested, and the mean value was recorded and used for analysis.

## Results and discussion

**Scheme 1:**
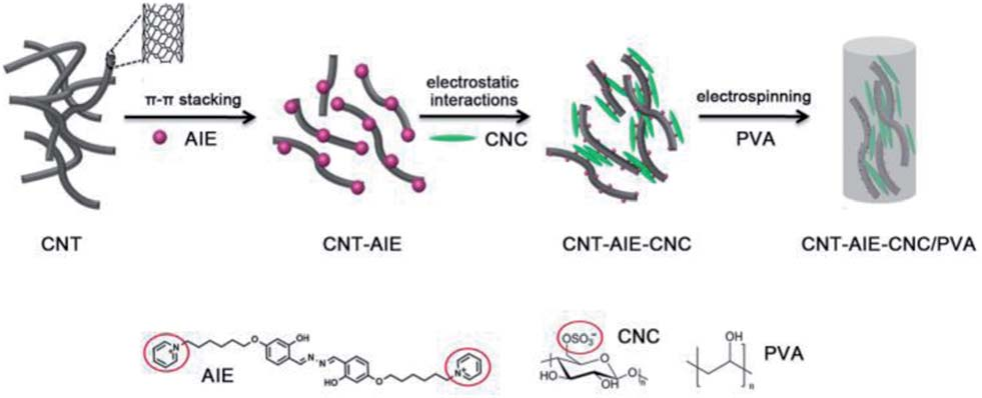
Design of the supramolecular ensemble of CNT–AIE–CNC as a filler for CNT–AIE–CNC/PVA.

The fabrication process for CNT–AIE–CNC/PVA electrospun fibers is illustrated in Scheme 1.

**Fig. 1:**
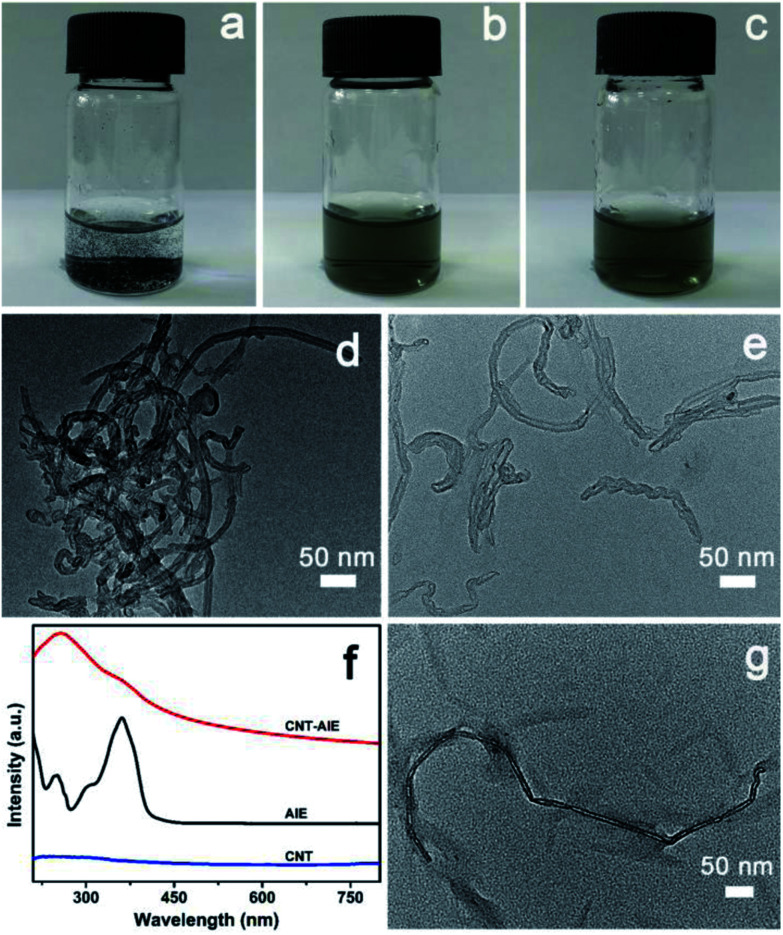
Tracking the formation of supramolecular ensemble of CNT–AIE–CNC. (a–c) Photographs showing transformation of heterogeneous mixtures to a homogeneous solution. (a) CNT in aqueous solution showing immiscible CNT. (b) CNT–AIE ensemble in CNT aqueous solution showing miscible CNT–AIE. (c) CNT–AIE–CNC in aqueous solution showing miscible CNT–AIE–CNC. (d) TEM image of pristine CNT in aqueous solutions. (e) TEM image of CNT–AIE in aqueous solutions. (f) UV-vis spectra. (g) TEM image showing the supramolecular ensemble of CNT–AIE–CNC.

Pristine CNT aggregated in aqueous solutions forming a heterogeneous mixture (Fig. 1a and d). The heterogeneous solution mixture turned homogeneous that remained stable after 24 h owing to formation of CNT–AIE supramolecular ensemble through π–π stacking (Fig. 1b). The TEM imaging analysis of the CNT–AIE suspension showed insignificant agglomeration of CNT (Fig. 1e). To further investigate the effect of AIE on the dispersion of CNT, UV-vis spectra were recorded for AIE, CNT and CNT–AIE suspensions. As shown in Fig. 1f, the CNT–AIE suspensions displayed a strong absorption peak at 258 nm and a shoulder peak at 360 nm corresponding to monodispersed CNT and AIE, respectively.^28,31^ It is worth taking note that the peak at 258 nm was absent in the UV spectrum of pristine CNT owing to severe agglomeration (Fig. 1f).^32^ The characteristic absorption peaks of AIE at 360 nm became less prominent in CNT–AIE. The aqueous solution of pristine AIE exhibited high fluorescence, while weak fluorescence was observed for an aqueous solution of AIE and CNT (Fig. S5†). This high degree of fluorescence quenching is owing to the formation of π–π stacking between AIE and CNT, similar to the reported π–π stacked aromatics-nanotubes.^33–35^ Based on spectral data, AIE was weakly emissive in dilute solutions but was highly emissive in the nanoaggregates of CNT–AIE that can be well retained in the electrospun fibers. Based on XPS analysis, the CNT–AIE ensemble contained around 39 wt% of AIE (Fig. S6†). Clearly, the formation of CNT–AIE ensemble prevents the agglomeration of CNT and increases the hydrophilicity, making it dispersible in aqueous solutions. Further addition of three times the mass of CNC, which was obtained from cotton pulp by sulfuric acid hydrolysis (ESI, Fig. S7†), to the CNT–AIE solution led to a soluble supramolecular ensemble of CNT–AIE–CNC owing to the electrostatic attractions between positively charged AIE and negatively charged CNC (Fig. 1c and g, S8†). Further characterization shows that the zeta potentials (*ζ*) of the aqueous suspensions of pristine CNT, AIE and CNT–AIE were −0.7 mV, 24.2 mV and 19.2 mV, respectively, showing that addition of AIE renders the AIE–CNT suspension positive charges. The *ζ* potential of 5 wt% CNC suspension was −21.3 mV, indicating the possibility of forming CNT–AIE–CNC through electrostatic attractions. The CNC nanorods have an approximate dimension of 10–20 nm in diameter and 200–300 nm in length based on TEM imaging analysis (Fig. S7†). The supramolecular ensemble of CNT–AIE–CNC with optimal CNT : CNC weight ratio of 1 : 3 was experimentally identified that showed significantly improved dispersion and less agglomeration in aqueous solutions based on high-magnification TEM image (Fig. 1g, S9†).

**Fig. 2:**
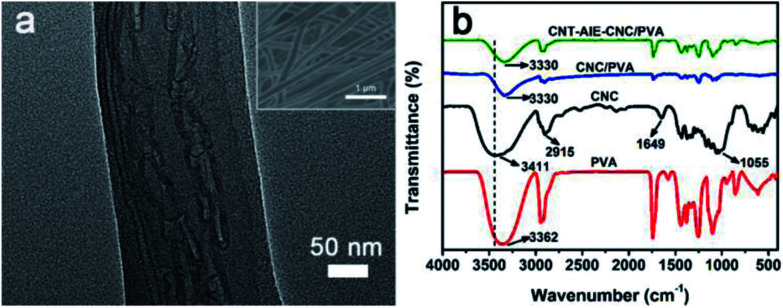
Characterizing CNT–AIE–CNC/PVA. (a) High magnification TEM image showing aligned and non-agglomerated supramolecular ensemble of CNT–AIE–CNC along fiber axis. Inset: low magnification SEM of CNT–AIE–CNC/PVA showing uniform fiber dimensions. (b) FTIR spectra of electrospun fibers of CNT–AIE–CNC/PVA and CNC/PVA at fixed filler : PVA weight ratio of 3 : 97 showing multiple hydrogen bonds between CNC and PVA.

The polymer nanocomposite fibers of CNT–AIE–CNC/PVA with the weight ratio of 3 : 97 was selected as a representative for discussion. The optimized fabrication condition of the polymer nanocomposite fibers was summarized in Table S1.† The fibers appeared to have smooth surface and uniform fiber dimension with a median diameter of 188.5 nm (Fig. S10g†). Notably, the CNT–AIE–CNC ensembles aligned along the fiber axis as clearly evidenced by the high-magnification TEM imaging analysis (Fig. 2a). The negatively charged CNC nanorods clustered around CNT–AIE through electrostatic interactions, which may contribute to straighten CNT leading to aligned configuration along the fiber axis. CNT–AIE–CNC and PVA further assembled by forming hydrogen bonds. For verification, CNT–AIE–CNC/PVA was characterized using FTIR spectroscopy. For comparison, the FTIR spectra of freeze-dried CNC, and the electrospun fibers of PVA and CNC/PVA were also recorded. As shown in Fig. 2b, the peaks at 3411 cm^−1^ and 2915 cm^−1^ were attributed to the O–H and C–H stretching vibration of CNC, and the peaks at 1649 cm^−1^ and 1055 cm^−1^ were assigned to the pyranose ring ether of cellulose and the O–H stretching vibration of absorbed water, respectively.^36^ It is evident that the characteristic peaks of –OH group of CNT–AIE–CNC/PVA shifted to lower frequencies, which are likely attributed to hydrogen bond formation between CNC and PVA.^12,37^ The presence of CNC in CNT–AIE–CNC/PVA is further indicated by the elemental analysis showing the presence of sulfur due to the sulfuric acid hydrolysis (Table S2†). It is interesting to note that the median diameters of polymer nanocomposite fibers increased from 176.5 nm, 188.5 nm to 204 nm with increasing (CNT–AIE–CNC) : PVA ratio from 1 : 99, 3 : 97 to 5 : 95 (Fig. S10†).

**Fig. 3:**
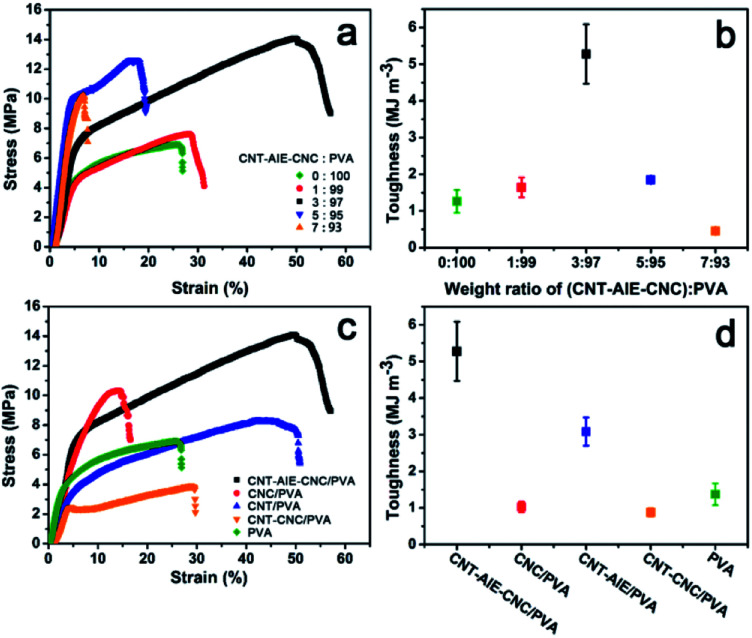
Characterizing the mechanical properties of CNT–AIE–CNC/PVA with varying weight ratio of (CNT–AIE–CNC) : PVA: (a) stress–strain curves. (b) Toughness. Comparing the mechanical properties of CNT–AIE–CNC/PVA with other filler : PVA electrospun fibers (CNC/PVA, CNT–AIE/PVA, CNT–CNC/PVA, PVA): (c) stress–stain curves. (d) Toughness.

The mechanical properties of CNT–AIE–CNC/PVA were characterized as shown in Fig. 3 and Table 1. It is clear from the measurements that the tensile strength of the polymer nanocomposite fiber was enhanced compared to the electrospun fibers of pure PVA, indicating the positive reinforcing effect of CNT–AIE–CNC. The degree of enhancement depended on the filler : polymer ratio, among which 3(CNT–AIE–CNC) : 97PVA performed the best showing significantly improved tensile strength (106%), elongation-at-break (104%), Young's modulus (35%) and toughness (308%). The reinforcement effect of CNT–AIE–CNC was further evaluated by comparing the mechanical properties of a series of filler/PVA electrospun fibers at the fixed filler : PVA weight ratio of 3 : 97. As shown in Fig. 3c, CNT–AIE–CNC/PVA outperformed those of CNC/PVA, CNT–AIE/PVA and CNT–CNC/PVA. Specifically, the tensile strength of CNT–AIE–CNC/PVA was higher by 38%, 71% and 267%, the corresponding elongation-at-break was higher by 264%, 19% and 76%, and the corresponding toughness was higher by 430%, 71% and 489%, respectively (Fig. 3b and d). It is clear that the synergistic effect among the fillers and at the filler–polymer interface enables stress transfer and resists fracture. During stretching, sliding between adjacent CNT may be partially resisted by the electrostatic attractions between CNC and CNT–AIE.^38^ It can be anticipated that further improvement in the mechanical properties can be approached by bridging CNT and CNC to allow more efficient load transfer.

**Table 1:** Mechanical properties of filler/PVA electrospun fibers

Electrospun fibers of filler/PVA	Filler content (wt%)	Elongation-at-break (%)	Tensile strength (MPa)	Young's modulus (MPa)
CNT–AIE–CNC/PVA	3	51	14.2	219
CNT–AIE–CNC/PVA	1	27	7.6	183
CNT–AIE–CNC/PVA	5	17	12.6	275
CNT–AIE–CNC/PVA	7	7	10.1	295
CNT–CNC/PVA	3	29	3.9	73
CNC/PVA	3	14	10.3	171
CNT–AIE/PVA	3	43	8.3	99
PVA	0	25	6.9	162

**Fig. 4:**
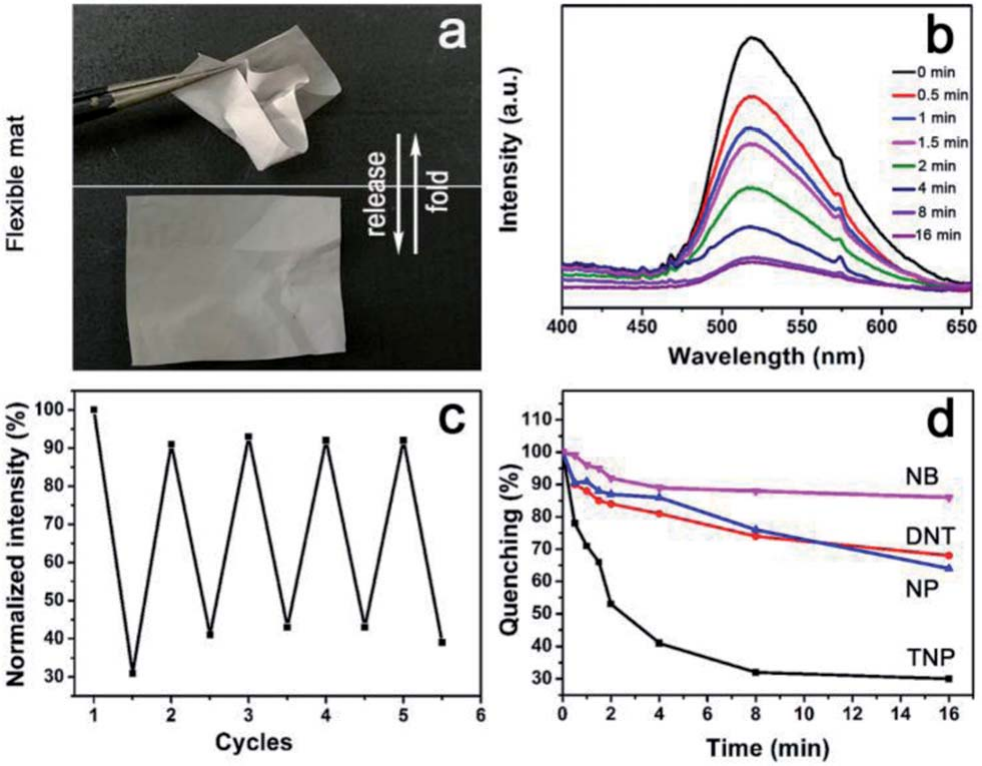
Characterizing the flexibility of the CNT–AIE–CNC/PVA mat. (a) Photographs showing the recoverability of the mat after folding. Characterizing the sensing ability of CNT–AIE–CNC/PVA for explosives. (b) Fluorescence quenching upon exposure to the saturated vapor of TNP (0–16 min). (c) Cycling test upon repeated exposure to the saturated vapor of TNP (16 min). (d) Time-dependent fluorescence quenching upon exposure to the saturated vapor of TNP, DNT, NP and NB, respectively.

We have demonstrated the ability of AIE to disperse CNT, and to bridge CNT and CNC through π–π stacking and electrostatic interactions, respectively, leading to water dispersible supramolecular ensemble of CNT–AIE–CNC that further assembles with PVA through hydrogen bonds forming CNT–AIE–CNC/PVA. AIE is sandwiched between CNT and CNC, and such a sandwiched ensemble is further compressed in the CNT–AIE–CNC/PVA fiber that may enable strong photoemission that may be used for developing optical sensing devices. CNT–AIE–CNC/PVA fiber mats have electron-rich surfaces due to the fluorogens of AIE. Studies show that electron-rich amine functionalized fluorescence donors enable optical response in exposure to electron-deficient aromatic rings through a photoinduced electron transfer and electronic energy transfer mechanism causing fluorescence quenching.^9,39,40^ For evaluation, free-standing and flexible fiber mats of CNT–AIE–CNC/PVA (filler : polymer weight ratio of 3 : 97) were examined for detection of nitroaromatic explosives (Fig. 4a). Taking 2,4,6-trinitrophenol (TNP) as a model, the detection was carried out by exposing the CNT–AIE–CNC/PVA fiber mat to saturated TNP vapor at room temperature (the saturation vapor pressures, orbital energies and saturated vapor concentration of the applied analytes are listed in Table S3†). As shown in Fig. 4b, the CNT–AIE–CNC/PVA fiber mat enabled strong photoemission under 360 nm irradiation. Upon exposure to the saturated TNP vapor, the photoemission intensity decreased gradually, that it reached 50% of the initial value in 2 min and 70% in 8 min (Fig. 4b and d). The quenching and recovery tests show that the photoemission of the CNT–AIE–CNC/PVA fiber mat can be restored to 92% of the initial value after 24 h of vacuum drying at 30 °C. The photoemission quenching upon exposure to TNP vapor reached around 62% after 16 min, suggesting reasonable reusability of the CNT–AIE–CNC/PVA fiber mat for explosive detection (Fig. 4c). The detection selectivity of CNT–AIE–CNC/PVA was further examined by exposing the fiber mat to the vapor of 2, 4-dinitrotoluene (DNT), 4-naphthalene (NP) and nitrobenzene (NB), respectively, at room temperature. As shown in Fig. 4d and S11,† the photoemission dropped intensity to 85% and 90% of the initial value in 2 min, and to 70% and 65% of the initial value in 16 min for DNT and NP, respectively. The photoemission intensity of the CNT–AIE–CNC/PVA fiber mat remained literally unchanged upon exposure to NB vapor. These results indicate that the CNT–AIE–CNC/PVA fiber mats are capable of selective detection of nitroaromatic explosives. The selective detection ability for TNP vapor is likely owning to its lower LUMO energy level (−3.92 eV) in favor of photoinduced electron transfer, compared to that of DNT (−2.96 eV), NP (−2.19 eV) and NB (−2.69 eV).^39,41,42^

## Conclusions

In conclusion, reinforcement by complementary elements in filler–polymer electrospun fibers such as CNT–AIE–CNC/PVA is the result of several mechanisms operating at the nanoscale. The degree of structural organization of CNT–AIE–CNC in the nanocomposite fibers afforded by the electrospinning process enables multiple interactions between the fillers and at the filler–polymer interfaces (π–π stacking, electrostatic interactions and hydrogen bonding) leading to improved energy dissipation. The presented results are proof-of-concept, and further studies are needed to optimize the interactions between the reinforcing fillers and the polymer phase, and to constrain the polymer chain motion that will ultimately result in highly efficient stress transfer path leading to maximized mechanical properties. The current study suggests a new strategy for rational design and organization of advanced polymer nanocomposite fibers from dissimilar and complementary elements for sensing and applications beyond.

## Conflicts of interest

There are no conflicts to declare.

## Supplementary Material

RA-008-C8RA01066H-s001
