# Supplementary material for: A supramolecular approach towards strong and tough polymer nanocomposite fibers
Source: RSC Adv. 2018 Mar 14;8(19):10361–6. doi: 10.1039/c8ra01066h (PMC9078929; doi:10.1039/c8ra01066h)
Supplement: RA-008-C8RA01066H-s001 [file RA-008-C8RA01066H-s001.pdf]

Electronic Supplementary Material (ESI) for RSC Advances.
This journal is © The Royal Society of Chemistry 2018

A Supramolecular Approach Towards Strong and Tough Polymer Nanocomposite Fibers

Xiaojuan Zhao, Hongzhi Zheng, Dan Qu, Haijing Jiang, Wei Fan, Yuyuan Sun and Yan Xu*

Preparation of cellulose nanocrystals (CNC)

In a typical experiment, 50 g of bleached commercial cotton pulp was milled using a commercial pulper containing 1000 mL of deionized water, followed by oven-drying. Next, 20 g milled pulp was hydrolysed in 200 mL of H_2SO_4 (1g pulp/10 ml H_2SO_4) aqueous solution (64 wt%) under vigorous stirring at 45 °C for 60 min. The pulp slurry was diluted with cold deionized water (about ten times the volume of the acid solution used) to stop the hydrolysis, and allowed to subside overnight. The clear top layer was decanted and the remaining cloudy layer was centrifuged. The supernatant was decanted and the resulting thick white slurry was washed three times with deionized water. Finally, the white thick suspension was placed into a Millipore ultrafiltration cell (model 8400) to wash the cellulose with deionized water until the pH of suspension was stable at a range of 6-7. The thick pulp slurry from the Millipore cell was diluted to desired concentration with constant stirring.

Preparation of AIE

Synthesis of compound 1. Synthetic route for AIE was demonstrated in Fig. S1. 2, 4-Dihydroxybenzaldehyde (690 mg, 5.0 mmol) and 1, 6-dibromohexane (2.4 g, 10 mmol) were first dissolved in acetone (20 mL), followed by addition of K_2CO_3 (690 mg, 5.0 mmol). The mixture was stirred at 60 °C in nitrogen for 24 h. After cooling to room temperature, the reaction mixture was extracted with dichloromethane (40 mL × 3). The combined dichloromethane fractions were dried over anhydrous $MgSO_4$ and concentrated under reduced pressure. The residue was further separated by column chromatography (silica, petroleum ether : ethyl acetate = 20 : 1) to give 1 as colorless oil (870 mg, 58% yield). Fig. S2 shows the ^{1}H NMR spectrum of 1 (500 MHz, DMSO), δ 10.99 (s, 1H), 10.00 (s, 1H), 7.62 (d, *J* = 8.7 Hz, 1H), 6.56 (dd, *J* = 8.7, 2.2 Hz, 1H), 6.47 (d, *J* = 2.2 Hz, 1H), 4.03 (s, 2H), 3.54 (s, 2H), 1.83 (s, 2H), 1.73 (s, 2H), 1.49 – 1.39 (m, 4H).

Synthesis of compound 2. Compound 1 (900 mg, 3 mmol) was dissolved in absolute ethanol (20 mL), followed by addition of hydrazine monohydrate (75 mg, 1.5 mmol). The mixture was refluxed for 8 h. Precipitates were filtrated under vacuum and washed with absolute ethanol three times to yield 2 as yellow powders after drying (715 mg, 80% yield). Fig. S3 shows the ^{1}H NMR spectrum of 2, (300 MHz, DMSO), δ 11.49 (s, 1H), 8.87 (s, 1H), 7.54 (d, *J* = 12.3 Hz, 1H), 6.53 (d, *J* = 16.0 Hz, 2H), 4.02 (s, 2H), 3.55 (s, 2H), 1.78 (d, *J* = 39.3 Hz, 4H), 1.42 (s, 4H).

Synthesis of AIE. Compound 2 (298 mg, 0.67 mmol) was dissolved in pyridine (4 ml, 50 mmol) and the mixture was stirred under reflux for 12 h. After completion of the reaction, the mixture was cooled to room temperature. The yellow solid product was separation by the suction filter and then the residue was recrystallized from a mixture of hexane/ethyl acetate to yield **AIE** as a yellow solid (430 mg, 85% yield). Fig. S4 shows the ^{1}H NMR spectrum of AIE, (300 MHz, DMSO) δ 11.49 (s, 1H), 9.13 (d, *J* = 5.5 Hz, 2H), 8.86 (s, 1H), 8.61 (t, *J* = 7.8 Hz, 1H), 8.23 – 8.11 (m, 2H), 7.53 (d, *J* = 8.8 Hz, 1H), 6.55 (dd, *J* = 8.7, 2.3 Hz, 1H), 6.50 (d, *J* = 2.3 Hz, 1H), 4.63 (t, *J* = 7.5 Hz, 2H), 4.01 (t, *J* = 6.3 Hz, 2H), 1.95 (s, 2H), 1.72 (s, 2H), 1.41 (d, *J* = 30.6 Hz, 4H).

Chemosensing experiments

Weighing bottles with the nitrocompound were placed in sealed glass flasks for 5 days to ensure that the equilibrium vapor pressure of the analytes is reached. Then, the electrospun nanofibrous films were brought into the glass flasks for a specific time period for exposure to the vapors. The emission spectra were recorded immediately by mounting the films on the sample holder for solids of the fluorescence spectrophotometer. The emission spectra of the nanofibrous films were recorded first for reference.

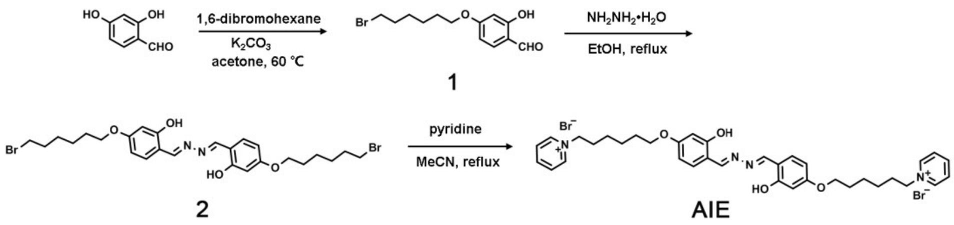

Fig. S1 Synthetic route for AIE.

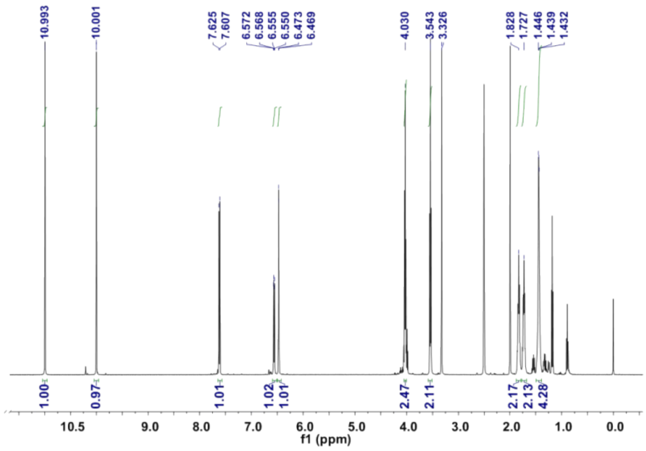

Fig. S2 ^{1}H NMR (500 MHz, DMSO, TMS, 25 ˚C) spectrum of 1.

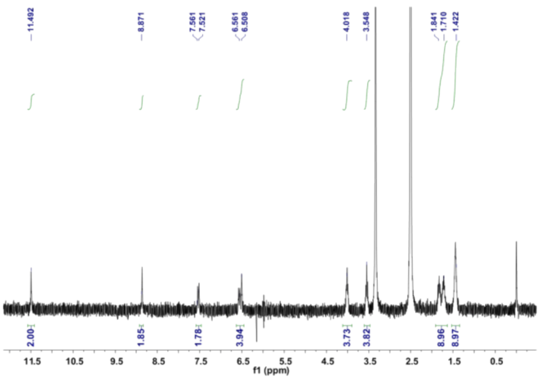

Fig. S3 ^{1}H NMR (300 MHz, DMSO, TMS, 25 ˚C) spectrum of 2.

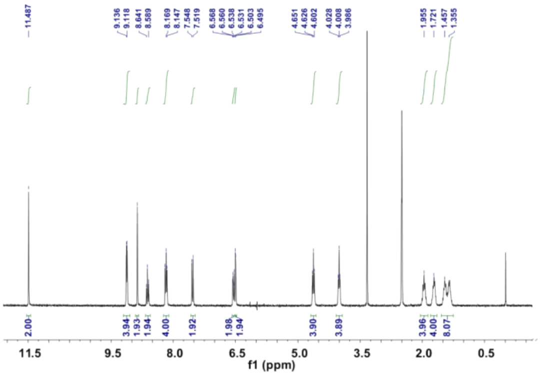

Fig. S4 ^{1}H NMR (500 MHz, DMSO, TMS, 25 ˚C) spectrum of AIE.

Table S1 The optimized fabrication condition of the polymer nanocomposite fibers.

Sample	PVA concentration	Spinning parameters			
		spinneret inner diameter (mm)	Voltage (kV)	Humidity (%)	Temperature (°C)
CNT-AIE-CNC/PVA	15%	0.51	14-16	30	20

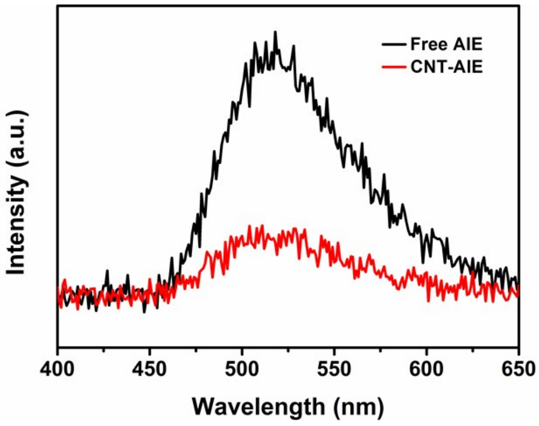

Fig. S5 Fluorescence spectra of solutions of free AIE and CNT-AIE with the same AIE concentration. Significant fluorescence quenching was evident for the formation of π-π stacking between AIE and CNT.

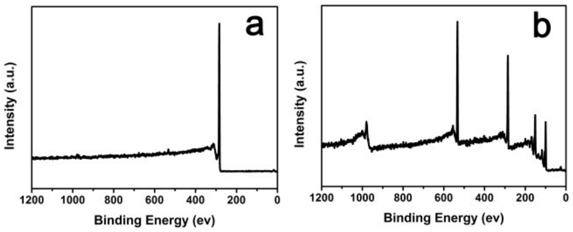

Fig. S6 (a) XPS spectrum of pristine CNT. (b) XPS spectrum of CNT-AIE.

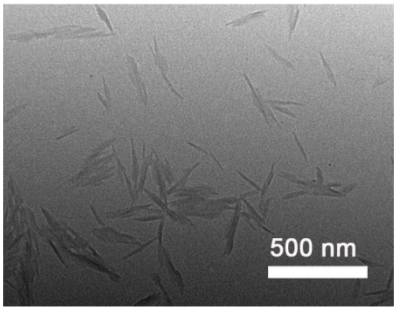

Fig. S7 TEM image of CNC nanorods.

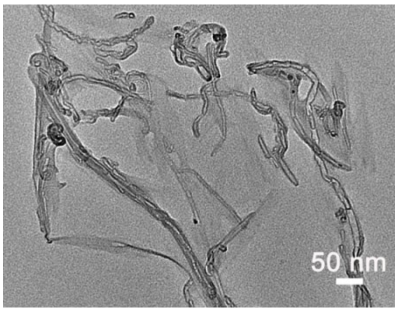

Fig. S8 Low magnification TEM image of the supramolecular ensemble of CNT-AIE-CNC.

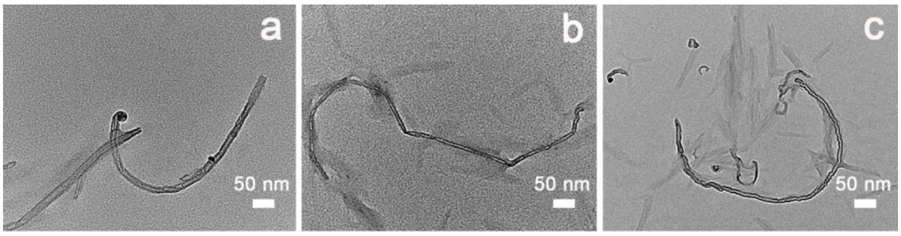

Fig. S9 Characterizing CNT-AIE-CNC with varying CNT:CNC weight ratio of (a) 1:1, (b) 1:3 and (c) 1:6.

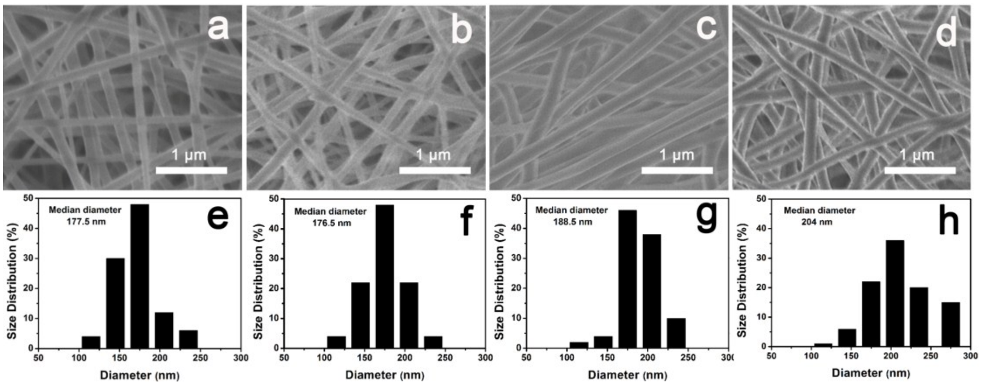

Fig. S10 Characterizing the electrospun fibers of CNT-AIE-CNC/PVA with varying weight ratio of (CNT-AIE-CNC):PVA at 0:100, 1:99, 3:97 and 5:95. (a-d) Corresponding SEM images showing the smooth surface. (e-h) Corresponding histograms showing the diameter distribution.

Table S2 Elemental analysis of CNC, PVA , CNC/PVA and CNT-AIE-CNC/PVA fiber.

Sample	N (%)	C (%)	H (%)	S (%)
CNC	0.000	41.68	6.159	0.720
PVA	0.000	52.03	8.348	0.000
CNC/PVA	0.000	47.79	7.862	0.440
CNT-AIE-CNC/PVA	0.100	51.33	8.397	0.246

Table S3 Vapor pressures, saturated vapor concentrations, and LUMO energies of analytes and interferences. Saturated vapor concentrations are calculated from vapor pressure at 25 ℃。

Compound	P_{vap} (mm Hg)	Vapor Concentration (ppb)	LUMO (eV)
TNP	5.80 E-9[1, 2]	7.63×10^{-3}	-3.92[3]
2,4-DNT	1.47 E-4[4]	1.93×10^{2}	-2.96[4]
4-NP	1.91 E-5[5]	2.51×10	-2.19[3]
NB	2.41E-1[2]	3.17×10^{5}	-2.69[6]

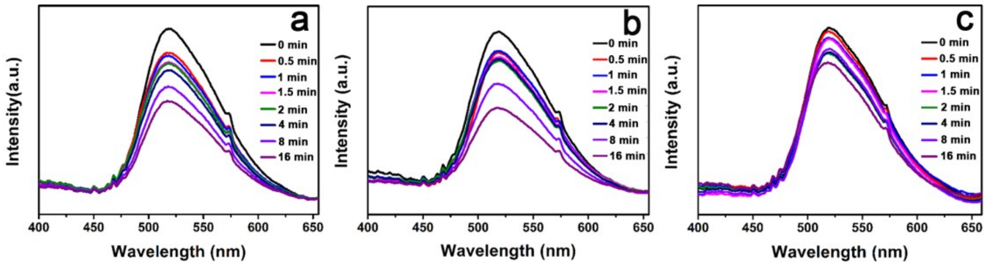

Fig. S11 Characterizing the sensing ability of CNT-AIE-CNC/PVA for explosives. (a) DNT. (b) NP. (c) NB.

Notes and references

1 B. Xu, X. Wu, H. Li, H. Tong and L. Wang, *Macromolecules*, 2011, **44**, 5089.

2 J. Qin, B. Ma, X.-F. Liu, H.-L. Lu, X.-Y. Dong, S.Q. Zang and H. Hou, *J. Mater. Chem. A*, 2015, **3**, 12690.

3 C. Gu, N. Huang, Y. Wu, H. Xu and D. Jiang, *Angew. Chem. Int. Ed.*, 2015, **54**, 11540.

4 Y. Wang, A. La, Y. Ding, Y. Liu and Y. Lei, *Adv. Funct. Mater.*, 2012, **22**, 3547.

5 M. K. Khaing Oo, Y. Guo, K. Reddy, J. Liu and X. Fan, *Analytical Chemistry*, 2012, **84**, 3376.

6 P. Ghosh, S. K. Saha, A. Roychowdhury and P. Banerjee, *Eur. J. Inorg. Chem.*, 2015, **2015**, 2851.
